# Comparative evaluation of biomedical and phytochemical applications of zinc nanoparticles by using *Fagonia cretica* extracts

**DOI:** 10.1038/s41598-022-14193-y

**Published:** 2022-06-15

**Authors:** Bushra Hafeez Kiani, Fizza Ikram, Humaira Fatima, Aiyeshah Alhodaib, Ihsan-ul- Haq, Tofeeq Ur-Rehman, Iffat Naz

**Affiliations:** 1 Department of Biological Sciences (Female Campus), Faculty of Basic and Applied Sciences, International Islamic University Islamabad, Islamabad, 44000 Pakistan; 2 Department of Pharmacy, Faculty of Biological Sciences, Quaid-I-Azam University, Islamabad, 45320 Pakistan; 3 Department of Physics, College of Science, Qassim University, Buraydah, 51452 Saudi Arabia; 4 Department of Biology, Science Unit, Deanship of Educational Services, Qassim University, Buraydah, 51452 Saudi Arabia

**Keywords:** Drug discovery, Plant sciences, Biotechnology, Nanobiotechnology, Plant biotechnology, Nanoparticles

## Abstract

The use of the green approach for nanoparticle synthesis yielded noticeable concern due to its eco-friendliness, cost-effectiveness, and reduced production of toxic chemicals. The current study was designed to formulate Zinc oxide nanoparticles (ZnO NPs) by using *Fagonia cretica* extracts, evaluating its phytochemical content, and different biological activities. Four different solvents; methanol (MeOH), n-Hexane (n–H), aqueous (Aq), and ethyl acetate (EA), had been utilized in the extracting method. ZnO NPs were successfully synthesized and characterized by UV–vis spectroscopy and scanning electron microscopy (SEM). The UV–vis spectra showed absorbance peaks between 350–400 nm range and SEM analysis revealed spherical morphology with particle sizes ranging from 65–80 nm. In phytochemical analysis, crude extracts exhibited the highest phytochemical content as they contain enriched secondary metabolites. n-hexane extract showed the highest phenolic contents while aqueous extracts showed the highest flavonoid content. Maximum free radicle scavenging activity was observed in NPs synthesized from ethyl-acetate extract with an IC_50_ value of 35.10 µg/ml. Significant antibacterial activity was exhibited by NPs polar solvents against *K. pneumonae*, *E. coli,* and *B. subtilis.* Polar solvents showed considerable antifungal potential against *A. flavus* and *F. solani*. NPs synthesized from nH extract showed potential cytotoxic activity with an LC_50_ value of 42.41 µg/ml against brine shrimps. A noteworthy antidiabetic activity was exhibited by nanoparticles synthesized from methanol extract i.e., 52.61 ± 0.36%. Significant bald zones were observed in nanoparticles synthesized from methanol extract rendering protein kinase inhibition. The present study highlights the significance of *F. indica* as a natural source for synthesizing functional nanoparticles with substantial antioxidant, antimicrobial, cytotoxic, protein kinase inhibitory, and antidiabetic properties.

## Introduction

From the beginning of human civilizations, plants are considered to be one of the oldest forms of human healthcare delivery known to date. Medicinal plants have diverse therapeutic potential hence used in different systems of medicine like Ayurveda, Allopathy, Unani, and Homeopathy^1,2^. According to World Health Organization (WHO), about 80% of the world’s population relies on traditional medicine for their prime health care needs^3^.

*Fagonia cretica* is one of the high medicinal value plants and it is of significant interest to pharmacists due to its efficient medicinal potential proved by preliminary pharmacological studies^4^. *Fagonia cretica* L. is a green, erect, minute, spiky bush of 1 to 3 feet in height mostly distributed in Algeria, Egypt, Tunisia, Cyprus, Morocco, Saudi Arabia, and dry calcareous rocks throughout western India and Pakistan^5,6^. *Fagonia* species are described to be curative having immense therapeutic effects against various conditions in scientific and folk medicine^7^. It is bitter and sour in taste, with enhanced medicinal significance against various foams of hepatic, hematological, neurological, and inflammatory conditions^8^. *Fagonia* species extracts are considered an antipyretic, antiasthmatic, antidote, antiseptic, antitumor, anti dysenteric, tonic, bitter, diuretic, analgesic, stomachic, and stimulant^9^.

Nanotechnology is one of the rapidly developing fields with many applications in diagnostics and therapeutic approaches. Due to their enhanced antimicrobial activity, NPs are considered as nano antibiotics^10^. At high temperatures and pressure these particles are more stable^11^. They contain mineral elements essential for the human body and therefore, some are recognized as non-toxic. In contrast to others the therapeutic potential of metal nanoparticles is relatively high^12,13^. These properties fascinated many researchers to discover innovative methods for the synthesis of different nanoparticles. Through conventional procedures less amount of time is required to synthesize a bulk amount of particles (physical and chemical methods), they require noxious chemicals such as defensive agents to nurture which contribute to toxic effects on surroundings. Using plants in the green approach is developing as an environment favorable, non-toxic, secure option. Biosynthesis of nanoparticles using plant extracts is cost-effective and also offers an innate capping medium in the pattern of protein^14^. Plant extract-mediated biosynthesis of nanoparticles of several metal NPs is utilized to manage synthetic toxic effects in the surroundings^15^.

Zinc oxide is very important in scientific research and industry as compared to other metal oxide nanoparticles, because of its exceptional features and widespread usage. It has exceptional optical, thermal, and chemical properties. Zinc Oxide nanoparticles find applications in sensors, catalysis, solar energy conversion, chemical storage, cosmetics, fibers, paints, microcapsule reactors, photoelectric material, and targeted drug delivery due to antibacterial and luminescence features^16^. ZnO NPs made from plant extracts are stable and diverse in form and magnitude as compared to those of other sources. Solvothermal synthesis, direct precipitation, reverse micelles, sol–gel method, homogeneous precipitation, sonochemical method, hydrothermal decomposition, microwave irradiation, and thermal decomposition are some of the methods used to produce these nanoparticles. The biological synthesis of nanoparticles is simple, environment friendly, and has wide antimicrobial action. The biosynthesis of ZnO nanoparticles was observed as a substitute to the chemical synthesis and less toxic to the atmosphere^17^. Green synthesis is environmentally affable, economical, and quick and the product does not have contaminants. In green synthesis, there is no need for precursors and NPs of diverse shapes and magnitudes are produced in bulk from plants. Usually, leaves and flowers are utilized frequently to synthesize zinc oxide nanoparticles^8^.

Metallic nanoparticles can be synthesized by using different methods i.e. chemical and biochemical synthesis which normally uses alkyl mercaptan, polyvinylpyrrolidone, dimethylformamide, thioanthracenol, or Tween 80 as the stabilizer and hydrazine hydrate sodium borohydride, or formaldehyde as the reducing agent to synthesize metallic nanoparticles^18^. These methods use toxic chemicals for the processing e.g. to maintain the stability of nanoparticles and are also very expensive. This threat to the environment because of these chemical methods researchers from all over the world focused on more reliable and eco-friendly methods for the synthesis of metallic nanoparticles using some natural sources.

Similarly, different chemical and physical methods are in use for the synthesis of ZnO NPs. These methods are very useful for the large-scale production of nanomaterials but they have adverse effects on the environment and human health due to the use of toxic chemicals^16^. Consequently, there is an urgent need for some eco-friendly and sustainable methods for the production of metal nanoparticles^19^. Therefore, some natural sources can be used as an alternative method for the production of ZnO NPs i.e. by using different microorganisms or different plant extracts to minimize the risk of environmental pollution^20,21^. Amid a variety of biosynthetic approaches, plant extracts are gaining global attention for the synthesis of various metal nanoparticles^22^. Polycyclic aromatic hydrocarbons (PAHs) are ubiquitous across the globe primarily due to persistent and long-term anthropogenic causes of pollution and are extremely persistent, and recalcitrant in the biosphere. PAH pollutants have been established to be highly mutagenic, toxic, carcinogenic, teratogenic, and immune-toxicogenic to various life forms. Different remediation methods involving physical, chemical, biological, and lately developed integrated approaches have been continuously applied with varying degrees of success. In this regard, recent studies have documented that ZnO NPs have shown great promise as an eco-friendly biological treatment solution for the remediation of PAHs^23^.

The focus of the present research was to explore and evaluate the biological and pharmaceutical properties of ZnO nanoparticles produced from aqueous, methanol, ethyl acetate, and n-hexane extracts obtained from aerial parts of the *Fagonia cretica.* Furthermore, the complete flavonoid and phenolic concentrations were also compared between crude extracts and ZnO NPs of different solvents. Enzyme inhibition assays i.e. protein kinase inhibition and α-amylase inhibition assays were also carried out to determine the efficacy of ZnO NPs against these enzymes in comparison with crude extracts.

## Results

### Extract recovery

To extract the phytoconstituents from *Fagonia cretica*, four solvents were used in a polar gradient approach. The total extract recovery was calculated for all the parts of the plant (Table 1).

**Table 1:** Extract recovery of *Fagonia cretica* samples.

S. No	Extract codes	Dielectric constant at 25 °C	Percent extract recovery % (w/w)
1	nH	1.88	1.90
2	EA	6.02	5.07
3	M	32.70	11.95
4	Aq	78.20	18.49

*nH n-Hexane, *EA* Ethyl Acetate, *M* Methanol, *Aq *Aqueous.

### Synthesis of zinc oxide nanoparticles

ZnO NPs were synthesized successfully by treating ethyl acetate, methanol, n-hexane, and distilled water extracts of roots and leaves with 0.01 M zinc acetate with constant stirring. After two hours of incubation, the color of the solution (pH 12) changed to off-white, which confirmed zinc nanoparticle synthesis. The solution was placed at 176°F for oven drying overnight and the pellet was oven-dried to get pure nanoparticles.

### Characterization of zinc nanoparticles

Scanning Electron Microscopy (SEM) and Ultraviolet–visible spectroscopy were used to analyze zinc nanoparticles.

### Ultraviolet–visible spectroscopy

**Figure 1:**
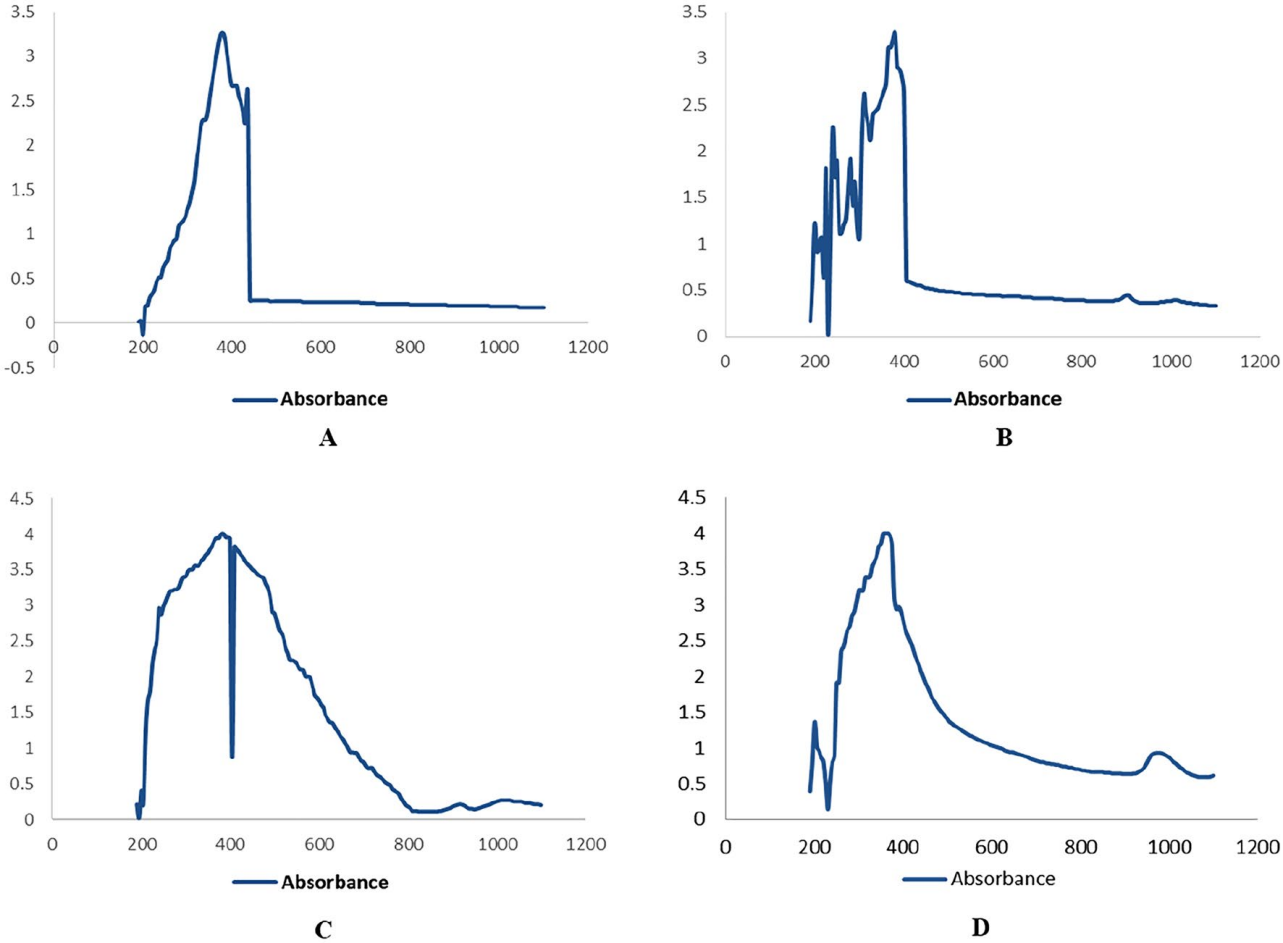
UV Visible absorption spectra of zinc oxide nanoparticles. (**A**) n-Hexane (**B**) Ethyl Acetate (**C**) Methanol (**D**) Aqueous.

Peaks for zinc nanoparticles between 350 to 400 nm were observed for the synthesized zinc oxide nanoparticles from the extracts of the aerial parts of the plant (Fig. 1).

### Scanning electron microscopy (SEM)

**Figure 2:**
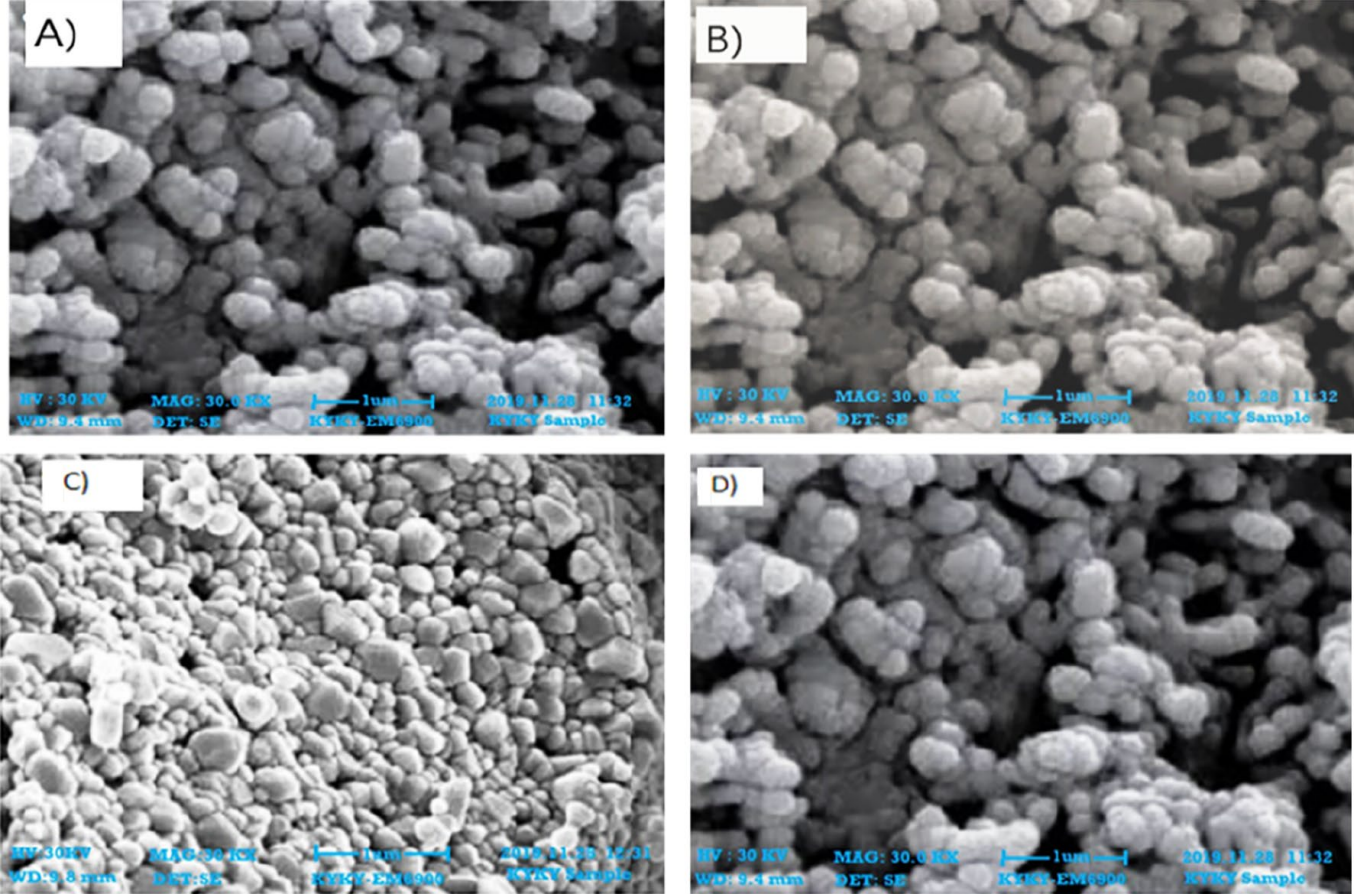
SEM images of zinc oxide nanoparticles. (**A**) n-Hexane (**B**) Ethyl Acetate (**C**) Methanol (**D**) Aqueous.

The SEM results are considered of great help to determine the morphology, size, and particle separation images of ZnO nanoparticles. It has been confirmed that the particles were within 100 nm for all four extracts of the aerial parts of the plants. The size was within the range of 65–80 nm at 30 kV for the synthesized ZnO NPs (Fig. 2). The shapes varied at some points but spherical was dominant and SEM analysis depicted the well scattered and combination of particles.

### Phytochemical analysis

#### Total phenolic contents

**Figure 3:**
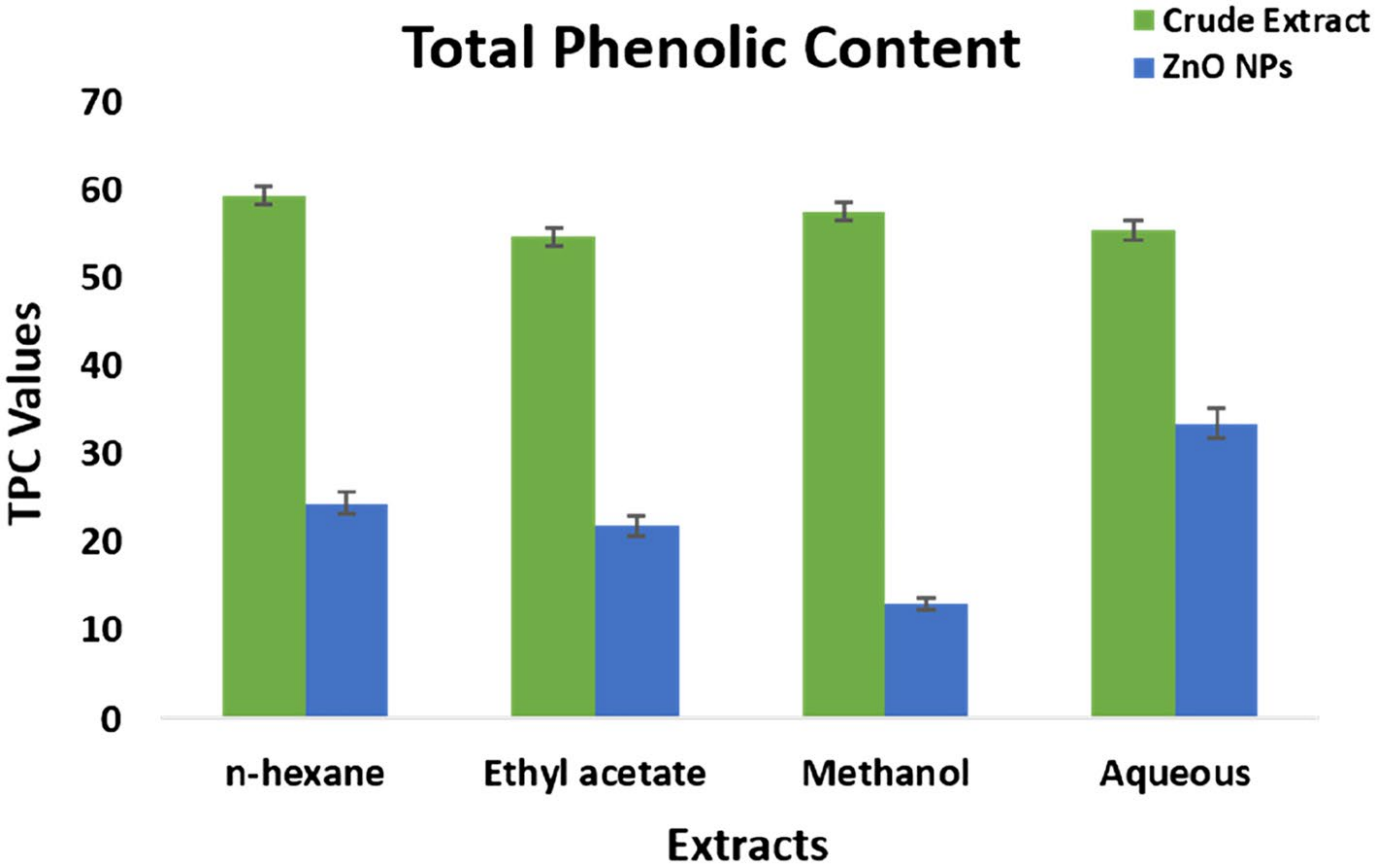
Total phenolic content of *F.cretica* crude extracts and its synthesized ZnO NPs. Values given are expressed as the mean of triplicate ± standard deviation.

Total phenolic content (TPC) showed the highest values in crude extracts as compared to nanoparticles (Fig. 3). A substantial effect was seen in the crude extracts. The maximum TPC was measured in crude extracts of n-hexane i.e., (59.32) followed by methanol (57.51), aqueous (55.41), and ethyl acetate (54.64). Nanoparticles showed minimum TPC as compared to crude extracts. For nanoparticles, the highest value was for aqueous (33.50) followed by n-hexane (24.41), ethyl acetate (21.92), and methanol (13.01).

#### Total flavonoid contents

**Figure 4:**
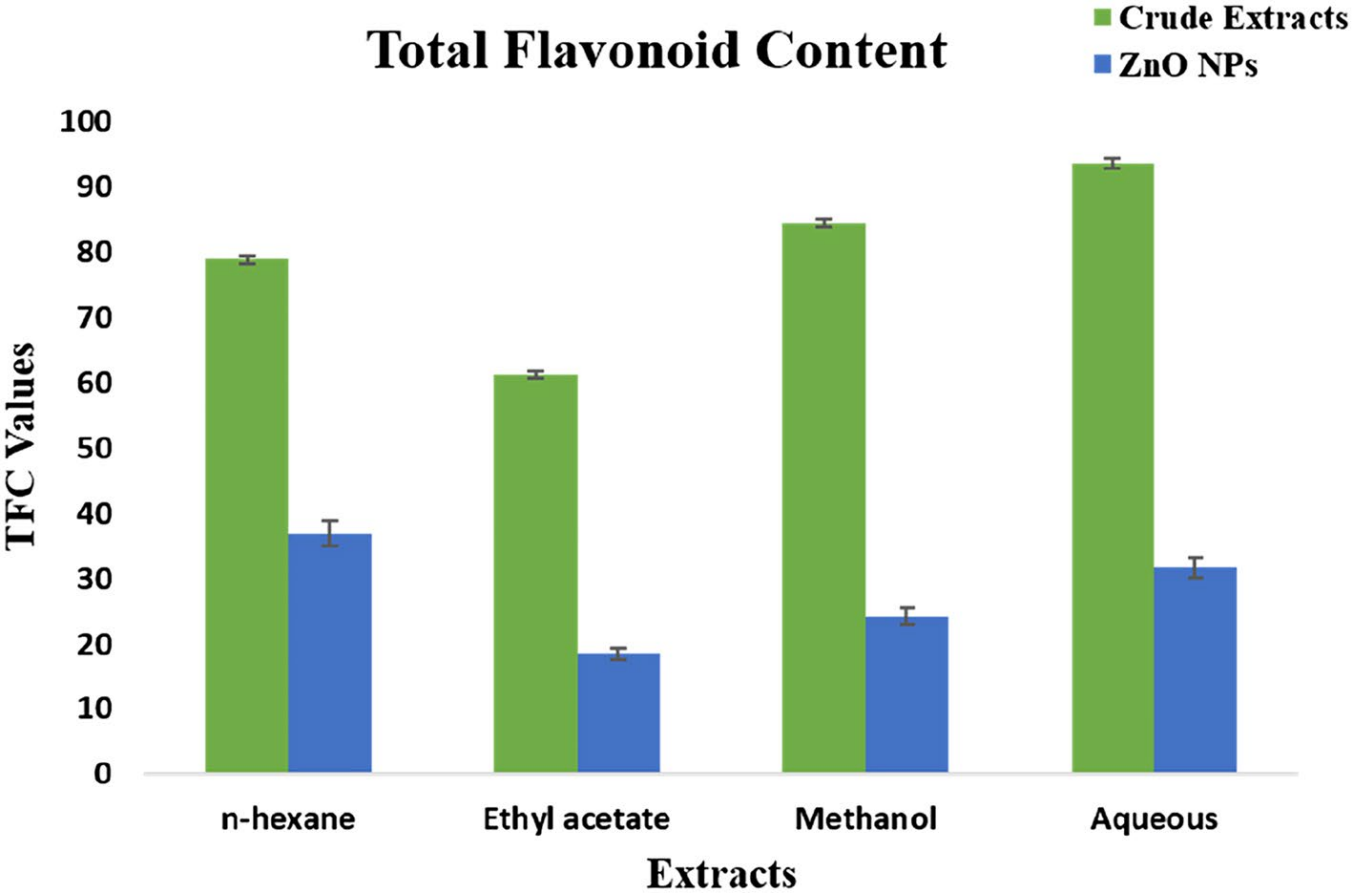
Total flavonoid content of *F.cretica* crude extracts and its synthesized ZnO NP. Values given are expressed as the mean of triplicate ± standard deviation.

The highest TFC was observed in crude extracts of the plant as compared to its synthesized ZnO NPs. The highest TFC was measured in aqueous extract i.e. 93.74 followed by methanol (84.49), n-hexane (78.87), and ethyl acetate (61.20). The synthesized nanoparticles showed minimum TFC as compared to crude extracts. For nanoparticles, the highest value was quantified in n-hexane (36.91) followed by aqueous (31.74), methanol (24.25), and ethyl acetate (18.52) (Fig. 4).

### Biological activities

#### Antioxidant assay

**Figure 5:**
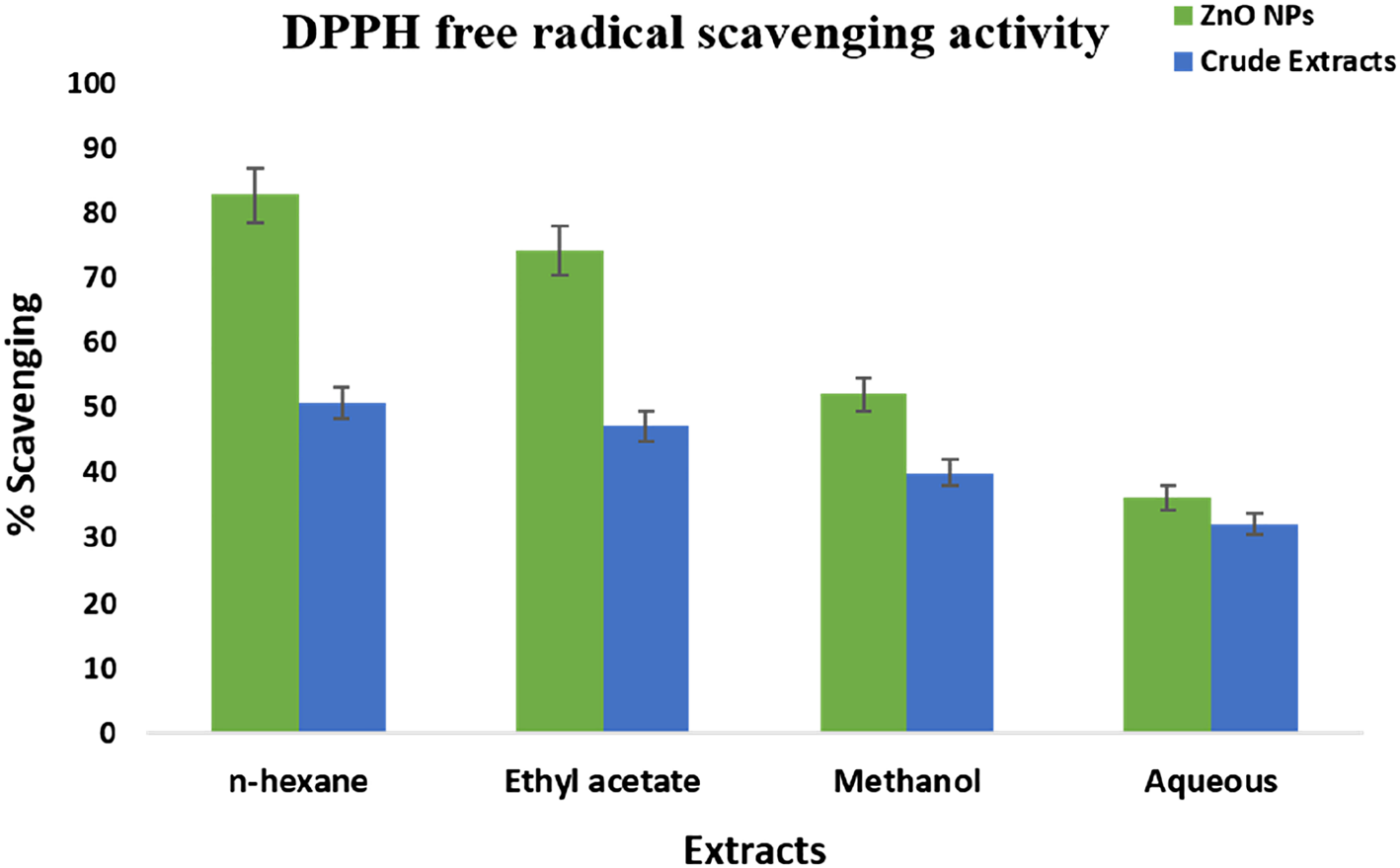
Comparative analysis of the antioxidant activity of ZnO NPs and crude extracts of *F. cretica.* Values given are expressed as the mean of triplicate ± standard deviation.

The free radicle scavenging activity was highest in nanoparticles as compared to crude extracts. For nanoparticles, n-hexane showed most promising activity with an IC_50_ value of 36.74, followed by ethyl acetate (IC_50_ = 35.10), methanol ((IC_50_ = 40.21)) and aqueous ((IC_50_ = 43.84). Crude extracts showed minimum free radicle scavenging activity (Fig. 5).

#### Antibacterial activity

**Figure 6:**
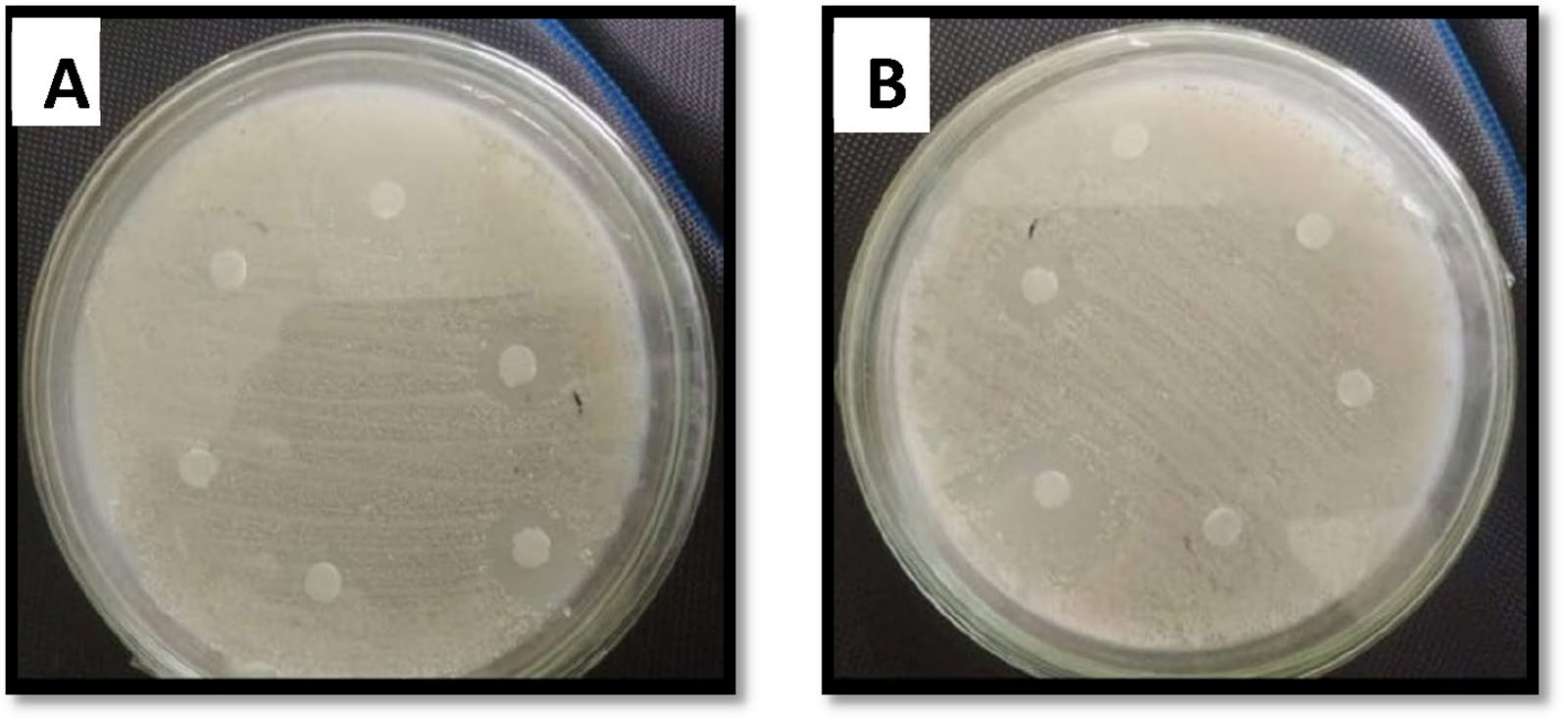
Antibacterial activity of *F. cretica* (A) Nanoparticles (B) Crude Extracts.

Antibacterial potential of *F.cretica* (aerial parts) crude extracts and the synthesize ZnO NPs were evaluated by using the disc diffusion methodology against a variety of strains of bacteria. In crude extracts, maximum activity was observed in methanolic extracts i.e., 14 ± 0.31 (MIC = 100 µg/ml) against *K. pneumoniae*, 12 ± 0.25 (MIC = 100 µg/ml) against *E. coli* and 12 ± 0.17 (MIC = 100 µg/ml) against *B. subtilis*. It was followed by aqueous i.e., 13 ± 0.27 (MIC = 100 µg/ml) against *B. subtilis*. No or least activity was observed against *S. aureus*, *P. aeruginosa*. For nanoparticles, considerable antibacterial activity was displayed by methanol i.e., 18 ± 0.19 (MIC = 33.3) against *K. pneumoniae*, 16 ± 0.23 (MIC = 100 µg/ml) against *E.coli* and 21 ± 0.40 (MIC = 3.7 µg/ml) against *B. subtilis*. This was followed by aqueous i.e., 15 ± 0.28 (MIC = 100 µg/ml) against *K. pneumoniae*, 13 ± 0.19 (MIC = 100 µg/ml) against *B. subtilis*. n-hexane showed antibacterial activity against *K. pneumoniae* 12 ± 0.25 (MIC = 100) and *E. coli* 12 ± 0.17 (MIC = 100 µg/ml), followed by ethyl acetate i.e., 12 ± 0.31 (MIC = 100 µg/ml) against *B. subtilis* (Table 2) (Fig. 6).

**Table 2:** Antibacterial activity and MIC values of *F. cretica* crude extracts and ZnO NPs.

Extract codes	Antibacterial assay									
	Diameter of zone of inhibition in mm (Mean ± SD) (MIC: µg/ml)									
	S.A	MIC	K.P	MIC	P.A	MIC	E.C	MIC	B.S	MIC
**Crude extracts**										
nH	7 ± 0.21	–	10 ± 0.25	–	5 ± 0.37	–	11 ± 0.17	–	7 ± 0.22	–
EA	7 ± 0.32	–	7 ± 0.17	–	5 ± 0.43	–	7 ± 0.21	–	6 ± 0.31	–
M	6 ± 0.25	–	14 ± 0.31	100	7 ± 0.21	–	12 ± 0.25	100	12 ± 0.17	100
Aq	8 ± 0.36	–	9 ± 0.27	–	6 ± 0.19	–	10 ± 0.28	–	13 ± 0.27	100
**Nanoparticles**										
nH	10 ± 0.19	–	12 ± 0.25	100	6 ± 0.21	–	12 ± 0.17	100	9 ± 0.22	–
EA	5 ± 0.36	–	9 ± 0.18	–	6 ± 0.37	–	7 ± 0.21	–	12 ± 0.31	100
M	9 ± 0.25	–	18 ± 0.19	33.3	9 ± 0.25	–	16 ± 0.23	100	21 ± 0.40	3.7
Aq	7 ± 0.32	–	15 ± 0.28	100	8 ± 0.19	–	8 ± 0.28	–	13 ± 0.19	100
DMSO	–	–	–	–	–	–	–	–	–	–

*The sample concentration was 100 µg per disc. Values (mean ± SD) are average of triplicate analysis of each plant extract (n value of 1 × 3). – = No activity. Samples showing zone of inhibition ≤ 12 mm were not proceeded for MIC determination. . S.A = *Staphylococcus aureus*, B.S = *Bacillus subtilis*, P.A = *Pseudomonas aeruginosa*, K. P = *Klebsiella pneumoniae*, E.C = *Escherichia coli.* . Positive control = Cef: Cefaxime, Rox: Roxithromycin. –- = no activity.

#### Antifungal assay

The antifungal potential of *F. cretica* (aerial parts) crude extracts and nanoparticles was evaluated by disc diffusion method. In crude extracts mild activity was shown by methanolic extract i.e., 11 ± 0.21 mm against *A. flavus*. The least activity against *F. solani* was shown by polar solvents’ crude extracts. No activity was observed against *A. fumigatus* and *Mucor*. For nanoparticles, the maximum activity was shown by polar solvent extract mediated ZnO NPs i.e., methanol against *A. flavus* (15 ± 0.40 mm) and *F. solani* (12 ± 0.31 mm). It was followed by aqueous extract; against *A.flavus* (13 ± 0.27 mm) and *F.solani* (9 ± 0.19 mm) respectively. No activity was observed against *A. fumigatus* and *Mucor* (Table 3).

**Table 3:** Antifungal activity of *F.cretica* crude extracts and its ZnO nanoparticles.

Plant name	Extract name	Sample	Antifungal activity			
			Zone of inhibition (mm ± SD)			
			*A. flavus*	*A. fumigatus*	*Mucor*	*F. solani*
*F. cretica*	nH	NP	7 ± 0.19	–	–	7 ± 0.25
		C	–	––	–	–
	EA	NP	–	–	–	–
		C	–	–	–	–
	M	NP	15 ± 0.40	–	–	12 ± 0.31
		C	11 ± 0.21	–	–	6 ± 0.11
	Aq	NP	13 ± 0.27	–	–	9 ± 0.19
		C	–	–	–	8 ± 0.21
	Control	Clot–	20 ± 0.57	20 ± 0.9	21 ± 0.57	22 ± 1.23

*DMSO = negative control. –- = no activity. Clot = clotrimazole.

Values (mean ± SD) are average of triplicate of each test sample (n value of 1 × 3).

#### Cytotoxicity activity

The cytotoxicity assay was performed using *F. cretica* (aerial parts) crude extracts and nanoparticles against brine shrimp larva. A significant cytotoxic effect was observed in crude extracts as compared to ZnO NPs. n-hexane extracts were more effective with LC_50_ value of 44.52 µg/ml, as compared to ethyl acetate with LC_50_ value 72.92 µg/ml. Methanol and aqueous extracts showed minimum activity with LC_50_ values ˃ 200 µg/ml. For ZnO NPs, n-hexane extract mediated ZnO NPs proved to be more active with LC_50_ value 42.41 µg/ml, followed by ethyl acetate extract mediated ZnO NPs with LC_50_ value of 62.45 µg/ml. The methanol extract mediated ZnO NPs (LC_50_:140 µg/ml) and aqueous extract mediated ZnO NPs (˃200 µg/ml) showed minimum activity (Table 4).

**Table 4:** Brine shrimp lethality potential of *F.cretica* crude extracts and its ZnO nanoparticles.

Plant name	Extracts	Samples	Percent mortality (%)			LC_50_ (µg/ml)
			200	100	50	
*F. cretica*	nH	NP	100 ± 1.50	100 ± 1.50	60 ± 1.0	42.41
		C	100 ± 1.50	100 ± 1.50	50 ± 1.40	44.52
	EA	NP	80 ± 1.25	70 ± 1.25	50 ± 1.70	62.45
		C	70 ± 1.15	60 ± 1.25	40 ± 1.0	72.92
	M	NP	60 ± 1.25	20 ± 1.60	10 ± 1.40	140.6
		C	40 ± 0.85	20 ± 1.0	–	˃200
	Aq	NP	20 ± 1.75	20 ± 1.50	–	˃200
		C	20 ± 1.75	10 ± 1.50	**–**	˃200

* Values given are expressed as a mean of triplicate ± SD. Positive control = Doxorubicin (5.93ug/ml). Negative control = DMSO.

* LC_50_ of positive control (Doxorubicin) was 5.93 µg/ml. DMSO was applied as negative control and the values given are expressed as a mean of triplicate ± SD.

### Enzyme inhibition assays

#### Protein kinase inhibition activity

**Figure 7:**
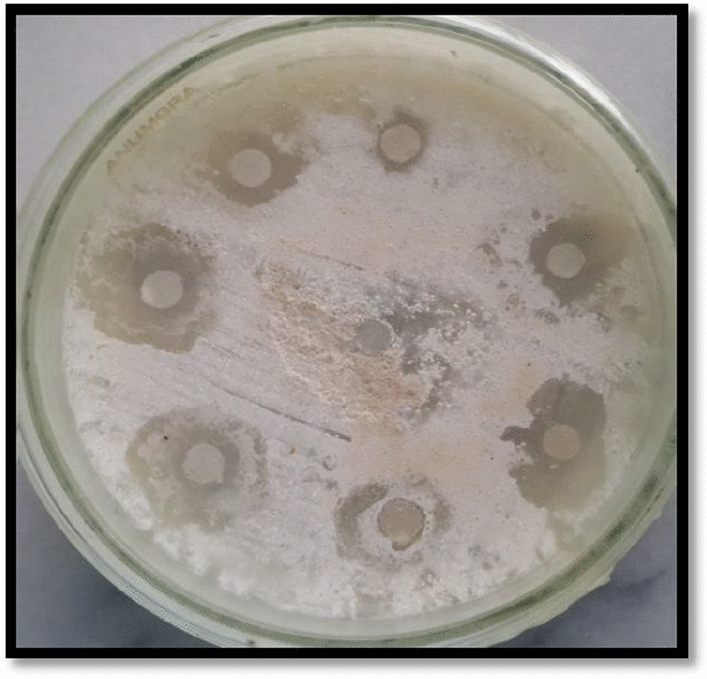
Protein kinase inhibition activity of *F. cretica* crude extracts and its ZnO nanoparticles. α-Amylase inhibition activity.

Among the crude extracts, a bald zone of inhibition was observed for methanol extract (10 mm). Ethyl acetate extract showed no inhibition (NA) while n-hexane and aqueous extracts showed clear zones i.e., 6 and 7 mm respectively. A moderate protein kinase inhibition was exhibited by the nanoparticles. Maximum bald zone of inhibition was observed by methanol extract mediated ZnO NPs (15 mm), followed by aqueous extract mediated ZnO NPs (11 mm), n-hexane extract mediated ZnO NPs (9 mm) and ethyl acetate extract mediated ZnO NPs (9 mm) (Fig. 7, Table 5).

**Table 5:** Protein kinase inhibition potential of *F. cretica* crude extracts and its ZnO nanoparticles.

Plant name	Extract name	Samples	Zones	Activity
*F. cretica*	nH	NP	9	Bald
		C	6	Clear
	EA	NP	9	Bald
		C	5	NA
	M	NP	15	Bald
		C	10	Bald
	Aq	NP	11	Bald
		C	7	Clear

*DMSO negative control, *Surfactin* positive control (20 µg/disc; 16 mm zone – = No activity.

**Figure 8:**
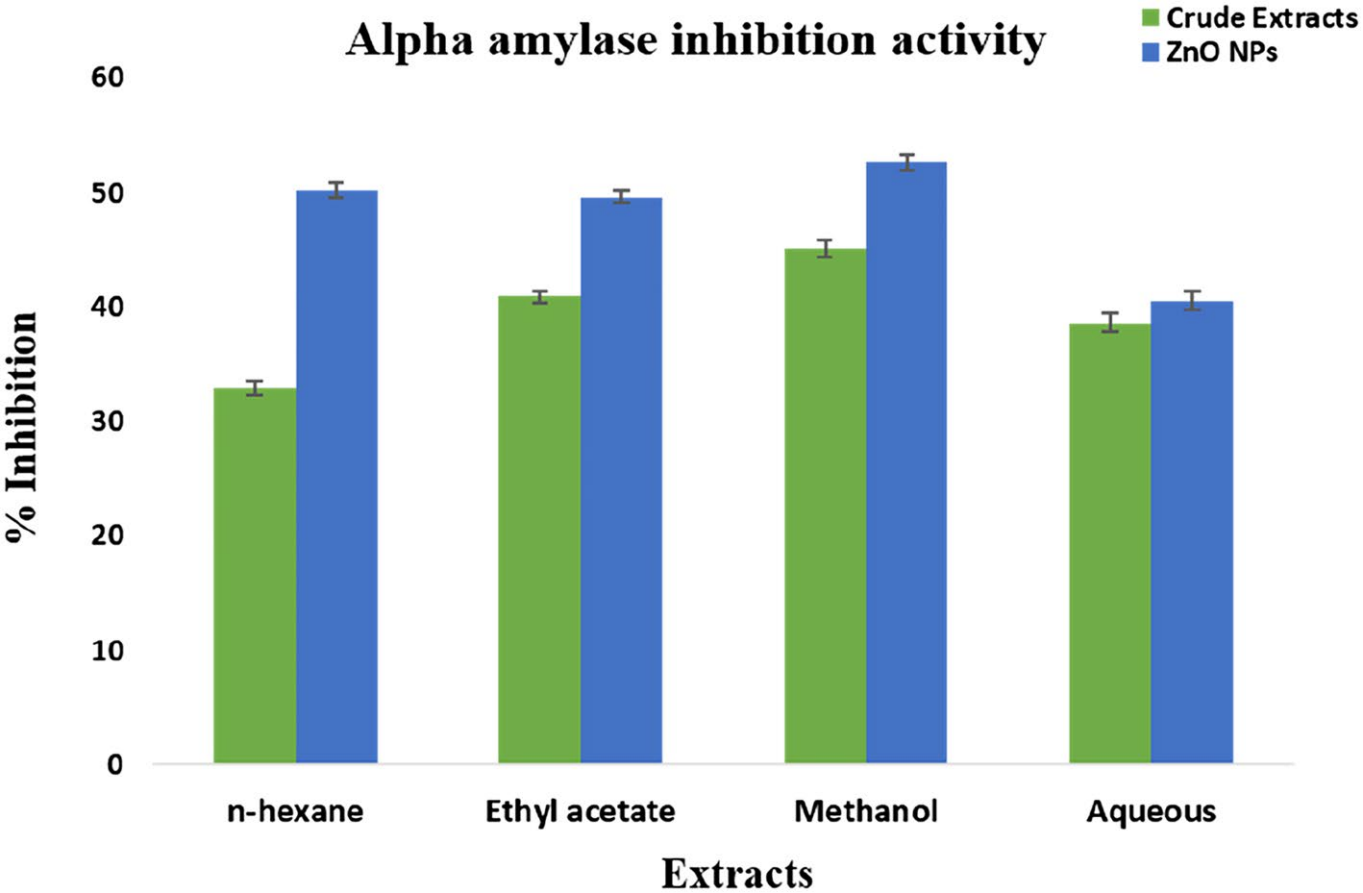
α-amylase inhibition potential of *F. cretica* crude extracts and its ZnO nanoparticles. Values given are expressed as the mean of triplicate ± standard deviation.

The α-amylase inhibition assay was performed using *F. cretica* (aerial parts) crude extracts and nanoparticles for the evaluation of their antidiabetic activity. Among the crude extracts, those obtained using methanol showed the highest activity i.e., (45.06 ± 0.19%), followed by ethyl acetate (40.88 ± 0.34%), n-hexane (32.90 ± 0.29%) and aqueous (38.57 ± 0.21%) crude extracts respectively. ZnO nanoparticles obtained from these extracts depicted different results from their crude extracts where maximum activity was exhibited in methanol extract mediated ZnO NPs (52.61 ± 0.36%). This was followed by ethyl acetate extract mediated ZnO NPs (49.60 ± 0.13%), n-hexane extract mediated ZnO NPs (50.22 ± 0.15%) and aqueous extract mediated ZnO NPs (40.53 ± 0.32%) (Fig. 8).

## Discussion

Throughout history, traditional medicine is considered the preferred primary health care system globally. Approximately 60% of the total world’s population and about 80% of developing countries rely on medicinal plants as a basic health care system. Herbal medicines have gained importance due to certain reasons including accessibility, efficacy, and affordability^24^. The therapeutic value of medicinal plants is relatively high because of the existence of bioactive phytochemical constituents. Fortunately, nature has gifted Pakistan with rich flora and its varied climate condition supporting the growth of nearly 6000 species of higher plants. Twelve and a half percent of plant species are reported for their medicinal values and their number is constantly increasing based on the interest of local researchers in natural products^6^.

Nanoparticles especially metallic nanoparticles have drawn attention in various fields like medicine, electronics, diagnostics, photonics, environment, and agriculture. Nanoparticle synthesis mediated by physical and chemical approaches is subjected to the toxic use of chemicals that have a higher risk of causing environmental toxicity and inducing carcinogenicity^25^. The synthesis of nanoparticles using biological entities as an effective method; gained significant importance by the use of microorganisms and plants residing medicinal value. However, the green approach for the synthesis of ZnO NPs is nontoxic, cost-reducing, and biocompatible^26^. ZnO NPs are extensively used in several commercial products as well as in biological and medical applications^27^.

*Fagonia cretica* is commonly known as ‘Dhamaasaa’and possesses a well-acknowledged medicinal stature. The subject plant turned out to be effective against fever, toothache, asthma, scabies, stomach troubles, tumors, and urinary discharges^28^ and also reported for its antimicrobial, anti-inflammatory, anti-hemorrhage, thrombolytic and antioxidant properties^9^. Very limited data is available on the synthesis of zinc nanoparticles using *F.cretica* plant extracts.

In the present study, ZnO NPs were synthesized first time using *F.cretica* extracts (aerial parts). A change in color i.e., to off-white, indicated the synthesis of ZnO NPs. UV and SEM characterized the synthesized nanoparticles. It is reported that UV–Vis spectroscopy can be used to examine the form and length of nanoparticles^29^. The absorption peaks for ZnO NPs were observed between 350–400 nm which are characteristics of ZnO nanoparticles. SEM analysis was used to determine the morphological attributes of the ZnO NPs. SEM analysis revealed the formation of fine, clear ZnO NPs, as well as an agglomeration of the particles, spherical in shape and particle size, ranging from 65–80 nm. These results are in accordance with the previous reports of^30^ and^31^ on the plant-based synthesis of ZnO NPs.

Phytochemicals are very important for the treatment of several degenerative abnormalities^32^. The results of phytochemical analysis and biological activities of *F. cretica* (aerial parts) crude extracts and nanoparticles revealed that high levels of flavonoids were found in n-hexane crude extracts as compared to nanoparticles (Fig. 3). The highest TFC values were measured in crude aqueous extracts in comparison with ZnO NPs (Fig. 4). The results of the present study are supported by^33,34^.

DPPH free radical scavenging activity results revealed that nanoparticles synthesized from ethyl acetate extract showed maximum scavenging activity i.e. 67.79% with IC_50_ of 35.10 µg/ml (Fig. 4) as compared to crude extracts. The crude extracts were unable to show notable free radical scavenging potential at optimized concentrations. Similar results were observed by^35–40^ while working with NPs of different plant species.

Alpha-amylase inhibition assay was performed for screening the anti-diabetic activity of *F.cretica* crude extracts and nanoparticles. α-amylase inhibition assay results showed that nanoparticles of methanolic extract showed high α-amylase inhibitory activity with 52% inhibition as compared to the crude extracts (Fig. 8). Zinc plays a prominent role in insulin action and carbohydrate metabolism Moreover, the antidiabetic activity of ZnO NPs synthesized from plants has also been reported by^41^ who found increased antidiabetic activity in nanoparticles. The crude extracts and synthesized ZnO nanoparticles of *F.cretica* were also screened for protein kinase inhibition potential. The results showed that nanoparticles synthesized from methanolic extract showed a remarkable bald zone of inhibition (Table 5, Fig. 7) in comparison with the crude extracts. The anticancer activity of ZnO nanoparticles has already been reported by^42^ in plants.

Antibacterial activities against *K. pneumoniae*, *E. coli*, *B. subtilis*, *S. aureus,* and *P. aeruginosa* have been evaluated. Samples that exhibited a significant zone ≥ 12 mm were subjected to MIC determination. The present study results showed that NPs displayed potential antibacterial activity against *K. pneumoniae* and *B. subtilis* (Table 2, Fig. 6). Our results are in accordance with the previously reported results of^42^. The crude plant extracts and synthesized nanoparticles were assessed for antifungal activity. Antifungal activity against *A. flavus*, *A fumigatus*, *Mucor*, and *F.solani* was investigated. The results showed that ZnO nanoparticles synthesized from polar solvent extracts showed significant antifungal activity against *A. flavus* and *F. solani* (Table 3) as compared to crude extracts. These findings are in contrast with the statement that plant extracts from non-polar solvents exhibit strong antimicrobial potential as compared to polar extracts^43^. Antifungal activities of ZnO NPs from different plants have been reported widely^42,44^.

Brine shrimp lethality assay has been thought of as an effective method for the evaluation of safety and toxicity profile of plant extracts and also determine their pharmacological activities^45^. The cytotoxic effect of *F. cretica* crude extracts and synthesized nanoparticles indicated that nanoparticles synthesized from n-hexane showed a substantial cytotoxic effect in comparison to the crude extracts (Table 4). These results are supported by a similar study on different green synthesized nanoparticles by^46^.

## Materials and methods

### Plant sample collection and identification

Fresh plants were collected from the Punjab province of Pakistan. An expert taxonomist Professor Dr. Mushtaq Ahmad at the Department of Plant Sciences, Quaid-i-Azam University, Islamabad, authenticated the plant as *Fagonia cretica* and its specimen preserved in the Department’s herbarium for future reference (ACC 543220). The aerial parts were separated and washed to clean debris and dried in shade. These dried parts were crushed with a pestle and mortar. The fine powder was stored separately for further use.

### Plant extract formulation

Extract of dehydrated *F. cretica* aerial parts was formulated through a simplified maceration process as explained by^47^. Four solvents, non-polar to the polar range, were used i.e., n-hexane (nH), ethyl acetate (EA), methanol (MeOH), and aqueous (Aq). For three days, 100 g of the powdered plant was soaked in 600 ml of each solvent. The soaked plant material was periodically sonicated at a 25 kHz frequency. After the specified period, filtration was done and re-extraction was done with the same solvent. Using a rotary evaporator, all filtrates from the respective solvents were mixed and left to dry. After being thoroughly dried, these crude extracts were kept at − 80 °C. Similar procedure was adopted for each solvent.

### Synthesis of zinc oxide nanoparticles (ZnO NPs)

The ZnO NPs were synthesized by utilizing the methodology of^48^ with few modifications. Plant extracts (50 ml) were heated in a beaker up to 50–60 °C on a hot plate for 30–40 min and 5 g of 0.01 M solution of zinc acetate (Sigma-Aldrich) was added directly to the heated extract. The mixture was constantly stirred for two hours at 50–60 °C on a hot plate. The color change was observed after 2 h which was the first visual confirmation of the synthesis of ZnO NPs and the extract was left to cool. The extract was transferred to Petri plates and spread as a very thin layer. The plates were left to dry overnight in a drying oven at 60 °C. The fine and dried powder was ready for the characterization procedure. The same process was repeated for each extract.

### Characterization of zinc oxide nanoparticles

#### UV–visible spectroscopy

UV–Vis spectroscopy is a widely utilized method for characterizing nanoparticles^49^. It adheres to the Beer-Lambert law^50^. The characterization of zinc oxide nanoparticles was done using a wavelength of 350–400 nm. The material was examined using a spectroscope, and the spectra were monitored between 300 and 700 nm with a 1-nm resolution.

#### Scanning electron microscopy analysis (SEM)

SEM (KYKY-EM6900) examination was utilized to examine the shape and size of nanoparticles on a micrometer to nanometer level^51^. Zinc oxide nanoparticles were evaluated by putting a droplet of sample solution onto a grid evenly covered with carbon, then dehydrating it beneath the mercury lamp for 15 min at 30 kV. It was examined and photographed. Finally, the instrument was equipped with an energy dispersive spectrum (EDS) to ensure nanoparticle presence.

### Phytochemical analysis

#### Total flavonoid concentration assessment

The complete flavonoid concentration was assessed as per the methodology of^52^. The extracts/samples (20 µl) were mixed with 10 µl potassium acetate, 10 µl aluminum chloride, and 160 µl of distilled water in 96 well plates. This mix was subsequently incubated for half an hour at room temperature. The absorbance was observed at a 405-nm wavelength on a microplate reader. To evaluate total flavonoid concentrations in equivalence to quercetin, the standard curve was constructed using quercetin solutions at values of 2.5–40 µg/ml. 20 µl of respective solvents were used as a negative control.

#### Total phenolic concentration assessment

Utilizing the Folin–Ciocalteu reagent, the complete phenolic content was measured according to the methodology of^52^. Plant extracts and standard solutions were made at a concentration of 1 mg/µl. A portion of 200 µl was loaded in a 96-well plate, along with 90 µl of Folin–Ciocalteu reagent, which had been stirred thoroughly. The solution was incubated at room temperature for 5 min before adding 90 µl of sodium carbonate and mixed thoroughly using a plate shaker. This resultant mixture was then incubated for a 60-min at room temperature before being measured using a microplate reader at a 630-nm wavelength. Gallic acid (3.125–25 µg/µl) was used for plotting the standard calibration curve. Gallic acid equivalents in percentage weight by weight were used to express the total phenolic content. As a negative control, 20 µl of the respective solvents were utilized.

### Biological activities

The following biological activities of *F. cretica* were performed.

### Antibacterial assay

The disc diffusion technique was used to analyze the antibacterial properties of each test extract in vitro as explained by^53^. Five bacterial strains i.e., two gram-positive bacteria namely *Staphylococcus aureus* (ATCC 6538) and *Bacillus subtilis* (ATCC 6633), and three gram-negative bacteria namely *Pseudomonas aeruginosa* (ATCC-15442), *Escherichia coli* (ATCC 15224), and *Klebsiella pneumoniae* (ATCC-1705) were used to analyze the antibacterial activity of plants extracts. On nutrient agar plates, a bacterial lawn was created using a fresh culture of strains of bacteria with a seeding density of 1 106 CFU/ml. Each test extract (5 µl from 20 mg per milliliter DMSO) was impregnated on sterile filter paper discs, with Cefaxime and roxithromycin (5 µl from 4 mg per milliliter DMSO) serving as positive controls and DMSO (5 µl) serving as the negative control. After 24 h of incubation at 37 °C, these discs were put on appropriately labeled seeded agar plates, and inhibition zones surrounding every disc were determined by measuring. This test was performed three times and the mean value was determined with the standard deviation.

The MIC was obtained using the technique described by^53^. The MIC of samples with significant inhibition zones, i.e. 12 mm, was determined using the micro broth dilution technique. Each strain of bacterial inoculum was made using a density (5104 CFU/ml) which was adjusted beforehand. In a 96-well plate, threefold sequential dilutions of every experiment sample were made utilizing nutrient broth up to final concentrations of 100, 33.33, 11.11, and 3.70 µg per milliliter. Bacterial cultures were rehydrated in broth culture for 11 h before being kept at 4 °C in the refrigerator.

### Antifungal assay

The antifungal test was performed according to the description of^53^. *Aspergillus fumigatus* (FFBP 66), *Mucor species* (FFBP 0300), *Fusarium solani* (FFBP 0291), and *Aspergillus flavis* (FFBP 0064) were all tested for antifungal activity. All fungal strains were cultured at 28 °C on 6.5 percent SDA (Sabouraud dextrose agar, pH 5.7) then stored in the refrigerator at 4 °C. The standard treatment was clotrimazole (4 mg/ml), while the negative control was DMSO. SDA plates holding 25 ml media were infected with 100 µl of fungal inoculum that had been renewed. On seeded SDA plates, sterile filter paper discs containing test extracts (5 μl, 20 mg/ml DMSO), DMSO (5 µl), and clotrimazole (5 μl, 4 mg/ml DMSO), had been inserted. These inoculated plates were placed for 24-h incubation at 30 °C, and the inhibition zones surrounding each disc were calculated in millimeters (mm).

### Brine shrimp cytotoxicity assay

In a narrow rectangle pan (22 × 32 cm) supplied with saltwater, brine shrimp (*Artemia salina*) eggs (Sera, Heidelberg, Germany) were spawned. To form two uneven portions, a 2-mm plastic separator with several holes were placed inside the pan. The eggs (about 25 mg) were dispersed inside the bigger section, which was shaded with aluminum foil while the other section was lighted. *Phototropic nauplii* (brine shrimp larvae) were collected by pipetting from the lighted side after being detached from their shells through the separator after one of the emergences.

The cytotoxicity experiment was carried out on a 96-well plate with different alphabets (A–H). 44 µl of seawater was poured into wells A and E of the microwell plate. Twenty-five microliters of seawater were poured into B, C D, F, G, H, and 6 µl of sample in A and E. 25 µl of the sample was taken from A and poured into well B, and from well B, 25 µl was added in well C and the same process was repeated for D, and 25 µl from D was discarded. This was done to ensure uniform dilution values. The same steps were repeated for E, F, G, and H and from H to discard. Ten shrimps were transferred into each well of the microplate and the quantity was completed by adding 300 µl seawater in all wells and kept for 24 h. The survival of larvae was observed under a microscope. The test was done three times, and Abbott's method was used to compute the percentages of dead larvae.

### Free radical scavenging property

The 2, 2, diphenyl-1-picrylhydrazyl (DPPH) test was used to determine the free radical scavenging property. The DPPH free radical test was performed using the methodology of^52^. DPPH of 9.6 mg was dissolved in 100 ml of methanol to make a solution of DPPH. The tested samples were prepared as 4 mg per milliliter in Dimethyl sulfoxide. Standard ascorbic acid was formed in DMSO as 1 mg/ml. In each well of a 96-well plate, an aliquot of 10 µl of the test material was introduced, accompanied by 190 µl of DPPH reagent. The mixtures were subsequently stirred and put for incubation at 37 °C for an hour in no light. Absorbance was calculated at 515 nm by an ELISA plate reader. DMSO was used as a negative control and ascorbic acid (ASA) as a positive control. The experiment was repeated three times for each test sample with IC_50_ values derived using table curves software, and percentage inhibition was computed using the following equation.

$$\% {\text{ DPPH}}\, = \,\left( {{1} - {\text{ Abs / Abc}}} \right)\, \times \,{1}00$$

where; “Ac” is Absorbance of negative control and “As” is Absorbance of the experimental sample.

### Enzyme inhibition assays

#### Protein kinase assay

In this assay, hyphae formation was observed in the purified strain of *Streptomyces* 85E according to the methodology designated by^54^. By distributing spores (mycelia fragments) from a fresh *Streptomyces* culture on sterilized plates with limited ISP4 media, a bacteria lawn was cultured. On sterilized 6-mm filter paper discs, about 5 μl of every extract (20 mg per milliliter of dimethyl sulfoxide) was poured. The impregnated paper discs were put directly on top of the plates inoculated with Streptomyces 85E at the peak ratio of 100 µg per disc. Discs injected with dimethyl sulfoxide and Surfactin were employed as negative and positive controls, correspondingly. These plates were put for a 3-day incubation at 30 °C (This is the time taken by *Streptomyces* 85E to produce hyphae), and the findings were assessed as a bald inhibition zone surrounding the samples and controls inserted discs.

#### α-amylase inhibition assay

The anti-diabetic capacity of sample extracts was assessed using the standard -amylase inhibition assay with slight modifications^55^. In a 96 well plate, a reaction mix comprising 25 µl of amylase (0.14 U/ml), 150 µl of phosphate buffer (pH 6.8), 40 µl of starch solution (2 mg per liter in potassium phosphate buffer), and 10 μl sample (4 mg per milliliter Dimethyl sulfoxide) was put for incubation at 50 °C for half an hour before being inhibited by 20 µl of 1 molar solution of hydrochloric acid. Following this, the individual well was filled with 90 µl of iodine solution (5 mM iodine, 5 mM potassium iodide). There were no extracts of plants in the negative control, while the blank was produced with no amylase and plant extract and both were substituted with equal amounts of the buffer. As a positive control, 250 μM acarbose was employed. After incubating, the absorbance of this reaction plate was assessed at 540 nm. The performance was measured in percent α-amylase inhibition per milligram dry extract. It was subsequently determined using this formula:

$$\% \alpha - - {\text{amylase inhibition }} = \, (Os - - On)/ \, (Ob - - On) \times { 1}00\%$$

where Ob = Blank well Absorbance, *Os* = Sample Absorbance, and *On* = Negative Control Absorbance.

### Ethics approval and consent to participate

No ethical considerations apply to this paper.

Experimental research on plants, including the collection of plant material complied with the relevant institutional, national, and international guidelines and legislation.

### Consent for publication

Not applicable.

## Conclusion

The present study reported a simple and successful synthesis of ZnO nanoparticles by using *Fagonia cretica* extracts. The resultant nanoparticles were characterized using UV–vis spectroscopy and Scanning electron microscopy (SEM). The UV–visible spectrum showed the characteristic peaks for ZnO nanoparticles ranging from 350–400 nm. SEM analysis disclosed that nanoparticles were spherical in appearance with particle dimensions ranging from 65–80 nm. Results of the present study revealed that the phytochemical content (phenols and flavonoids) of pure plant extract is higher than those of synthesized nanoparticles. Moreover, the study demonstrates that ZnO nanoparticles synthesized from *F. cretica* extract exhibit strong antibacterial, antifungal, antioxidant, antitumor, antidiabetic, and cytotoxic activities. Biocompatible nanoparticles synthesized by employing plant (enriched pharmacological active compounds) have diverse applications as nanomedicines in pharmaceuticals, targeted drug delivery, food, cosmetics, and agriculture, thus becoming major candidates in biomedical research. In conclusion, the results of the undertaken study show that eco-friendly ZnO NPs synthesized from ethno-medicinally valued *F. indica* could be used biomedically for the treatment of anomalies allied with oxidative stress, infections, and as an antidiabetic. Further studies on the isolation of active compounds of this plant can be planned to discover new drugs.

## Data Availability

The datasets generated during and/or analyzed during the current study are available from the corresponding author on reasonable request.
